# Adolescent distinctions between quality of life and self-rated health in quality of life research

**DOI:** 10.1186/1477-7525-3-64

**Published:** 2005-10-25

**Authors:** Keith J Zullig, Robert F Valois, J Wanzer Drane

**Affiliations:** 1 Health Education, Department of Physical Education, Health, & Sport Studies, Miami University, Oxford, OH 45056, USA; 2 Arnold School of Public Health, Department of Health Promotion, Education & Behavior, University of South Carolina, Columbia, SC 29208, USA; 3 School of Medicine, Department of Family & Preventive Medicine, University of South Carolina, Columbia, SC 29208, USA; 4 Arnold School of Public Health, Department of Biostatistics & Epidemiology, University of South Carolina, Columbia, SC 29208, USA

## Abstract

**Background:**

In adult quality of life (QOL) research, the QOL construct appears to differ from self-rated health status. Although increased QOL continues to be recognized as an important outcome in health promotion and medical intervention, little research has attempted to explore adolescent perceptual differences between self-rated health and QOL.

**Methods:**

Correlational analyses were performed between self-rated health, physical health days and mental health days, and QOL. Data were collected from two different public high school adolescent samples during two different time periods (1997 & 2003) in two different geographic regions in the USA (a southern & midwestern state) with two different sample sizes (N = 5,220 and N = 140, respectively) using the CDC Youth Risk Behavior Survey (YRBS). The Centers for Disease Control and Preventions' health-related quality of life scale (HRQOL) provided estimates of self-rated health, physical health days and mental health days, and QOL.

**Results:**

All correlation coefficients were significant in both samples (p ≤ .0001), suggesting sample size was not a contributing factor to the significant correlations. In both samples, adolescent QOL ratings were more strongly correlated with the mean number of poor mental health days (r = .88, southern sample; r = .89, midwestern sample) than with the mean number of poor physical health days (r = .75, southern sample; r = .79, midwestern sample), consistent with adult QOL research. However, correlation coefficients in both samples between self-rated health and the mean number of poor physical health days was slightly smaller (r = .24, southern, r = .32, midwestern) than that between self-rated health and the mean number of poor mental health days (r = .25, southern, r = .39 midwestern), which is contrary to adult QOL research.

**Conclusion:**

Similar to adults, these results suggest adolescents are rating two distinct constructs, and that self-rated health and QOL should not be used interchangeably. QOL, in the context of public high school adolescents, is based largely upon self-reported mental health and to a lesser extent on self-reported physical health. Conversely, although self-reported mental health and self-reported physical health both contribute significantly to adolescent self-rated health, mental health appears to make a greater contribution, which is contrary to observations with adults. Health promoting efforts for adolescents may need to focus more on mental health than physical health, when considering population needs and type of micro or macro intervention.

## Background

It has become accepted that increasing one's quality of life (QOL) via health promotion efforts and medical care interventions is a desirable outcome for both adolescents and adults [1-3] and monitoring adult QOL continues to be of interest in the United States [4,5]. Monitoring adolescent QOL is also beginning to receive attention in some adolescent literature [6,7].

Although definitions vary, QOL has been defined as "a popular term that conveys an overall sense of well-being, including aspects of happiness and satisfaction with life as a whole. "It is broad and subjective rather than specific and objective" [[8], p.5]. Through this definition, self-rated health status is viewed as an important domain for overall QOL, but how important self-rated health status is in regard to QOL has been difficult to quantify, partly because prior research has not adequately defined what QOL means to individuals. For example, Gill and Feinstein [9] found in their literature review, researchers seem to switch QOL with other terms such as "health status" or "functional status" in their definitions. Further complicating the definition of QOL is the term health-related QOL (HRQOL).

McHorney [10] suggests HRQOL has evolved to encompass those aspects of overall QOL that can be clearly shown to affect health – either physical or mental. This is supported by Carr et al. [11] who suggest that while QOL encompasses those aspects of an individual's subjective experience that relate both directly and indirectly to health, disease, disability, and impairment, "HRQOL is the gap between our expectations of health and our experience of it." (p.1240). More succinctly, the Centers for Disease Control and Prevention (CDC) defined HRQOL as "an individual's or group's perceived physical and mental health over time" [[8], p8]. However, in attempting to address the conceptual confusion between QOL, HRQOL, and self-rated health status, Smith, Avis, & Assmann's [12] conducted a meta-analysis of 12 chronic disease studies and concluded "that only two domains – mental health and physical functioning – are key determinants of QOL judgments" (p.457). Thus, for clarification purposes in this paper, QOL is defined using those two domains: perceived physical and mental health days. Nevertheless, it is important to define self-rated, of which its importance has been delineated in a number of resources and deserves further attention.

### QOL and self-rated health status

An extensive body of literature exists in regard to self-perceived, rated, or assessed health, particularly in reference to its predictive ability of morbidity and mortality as more detailed health status indicators for both adolescents and adults [13-24]. When asked "Would you say your health is excellent, very good, good, fair, or poor?" or a variation thereof, a significant association has been established with the risk of mortality over a four to nine-year period [16,17,24] and with risk behaviors such as smoking, exercise, sleep, body weight, and alcohol consumption in adults [13] and with personal, socio-environmental, behavioral, and psychological factors (e.g., health problems, disability, age, female gender, income, smoking, and higher BMI) in adolescents [20-22]. In addition, recent evidence suggests a measure of self-rated health was able to discriminate between risk factors and diabetes care among adolescents with Type I diabetes [23]. For example, male gender, higher parental socioeconomic level, a younger age of diagnosis, shorter diabetes duration, an no hospitalization in the preceding 6 months were all associated with better self-rated health.

As noted by Smith et. al [12] when clarifying the distinction between QOL and self-rated health status, these authors noted when rating QOL, patients give much greater emphasis to mental health (r = 0.47) than to physical functioning (r = 0.28). However, this pattern is reversed for appraisals of self-rated health in which physical functioning is more important than mental health. Their study provides evidence that adults are evaluating two different constructs. Their model also included social functioning, yet only weak correlations were established between QOL (0.14) and self-rated health (0.11). When social functioning was removed from the model, the contribution of mental health to QOL was 1.6 times as large as the contribution of physical functioning, suggesting perhaps the impact of social isolation or small social networks on the mental health of individuals. In other words, social functioning may be reflective of an individual's mental health, which in turn has greater effects on QOL than it does on self-rated health.

The finding that social functioning may overlap with mental health is an important finding when attempting to measure QOL, particularly among adolescents. It has been argued that in addition to measuring physical and mental health, adolescent QOL measurements should contain a social health component as well [14,25-27]. Socialization may be viewed as an important component for adolescents as they attempt to "fit in" among their peers, but it may not be as important when attempting to measure QOL. For example, Eisen, Ware, Donald, & Brook [28] found more overlap than expected between social and mental health in the RAND Corporation Health Insurance Study (HIS) of adolescents. They concluded:

"Whether the HIS social relations items are indicative of social health is open to question; they may instead be assessing a positive aspect of mental health. Although further study is required to clarify this issue, these analyses suggest either that HIS social relations items may not adequately measure the social component of child health or that mental and social components of child health are more substantially interrelated than hypothesized" [[28], p.919].

Therefore, it appears that social functioning may not be as important a determinant for QOL for either adults or adolescents. However, further investigation is warranted in the study of adolescent QOL and self-rated health status and begs the question as to whether QOL and self-rated health status measures are viewed as the same construct by adolescents, or do they represent differing constructs, as has been determined among adults? Furthermore, does mental health play a larger role for adolescent QOL and self-rated health ratings? Existing evidence suggests rising rates in adolescent conduct problems, depression, and suicide in nearly all developed countries since the Second World War [29,30]. Moreover, these trends are observed both among females and males, in all social classes, and among all family types [31]. Among American adolescents, Zullig et al. [32] found that although 60–62% of adolescents reported one or more poor physical or mental health days, respectively, in the past 30 days, as the number of reported poor health days increased to six or more days, poor mental health days significantly outweighed the number of poor physical health days (23% vs. 11% days, respectively). In addition, elevated levels of poor mental health days may last until about age 24 before declines are observed [5]. In light of these observations, it may be that adolescents give greater emphasis to mental health when reporting both their QOL perceptions and self-rated health.

Therefore, the purpose of this study was to examine the relationships between adolescent self-rated health, physical health, mental health, and QOL. Specifically, we seek to answer the question of whether mental health is more salient in both adolescent QOL and self-rated health ratings when compared physical health. Like adults, we hypothesized adolescent QOL would be more strongly related to mental health than physical health, but unlike adults, adolescent self-rated health would also be more strongly related to mental health than physical health. This research could have important implications for health practitioners, medical personnel, and researchers working with clinical populations, because when a measure of self-rated health is potentially used to assess QOL, the findings could be misleading. Bradley [33], for example, makes the distinction that asking participants how they feel about their health is different from asking them how they perceive their quality of life because, although people may feel their health is poor, their quality of life may be excellent or vice versa. Thus, efforts to achieve excellent health may actually damage QOL. Therefore, questioning self-rated health alone can have a confounding effect.

## Methods

This study used data collected from two different adolescent samples at two different time periods in two different geographic regions in the USA to provide multiple indicators of model validity. The first sample of public high school adolescents originated in a southern state via the 1997 CDC-Youth Risk Behavior Survey (YRBS). The second sample utilizes a randomly selected sample of public high school adolescents in a Midwestern state and was collected in 2003. The Midwestern state adolescent data were collected as part of another program evaluation using the 2003 YRBS in the same fashion as the southern state sample.

### Instrumentation

The core CDC health-related QOL scale was used for this study because it contains a measure of self-rated health and measures of physical and mental health days, both of which have been determined to be the key components of QOL among adults [12]. The CDC scale is based on research with adults age 18 or greater and initially began with the 4 core questions on the Behavioral Risk Factor Surveillance System (BRFSS) in 1994 [34,35]. Item 1 focuses on self-rated health "In general, how would you rate your health?" Consistent with previous research, response options for this item are excellent, very good, good, fair, and poor. Items 2 and 3 relate to recent physical and mental health symptoms, are considered mutually exclusive, and are worded as such "Now thinking about your physical (or mental) health, for how many days during the past 30 days was your physical (or mental) health not good?" Item 4 is conceptualized as a global measure of disability that explicitly incorporates both physical and mental health: "During the past 30 days, on how many days did poor physical or mental health keep you from doing your usual activities...?" For the purposes of this study, this item was omitted from all analyses.

All response options to the scale "days" items were identical and assessed the number of days symptoms were experienced: 0 days, 1–2 days, 3 to 5 days, 6 to 9 days, 10 to 19 days, 20 to 29 days, and all 30 days. This scale was determined to be valid among adults [36-38] and recently in a large, randomly selected population of adolescents through paper and pencil administration [32]. Two telephone-based reliability studies have also been conducted on the scale, revealing considerable test-retest reliability [39,40]. In both samples, this 4-item scale was amended to the end of the standard YRBS, eliminating any potential instrumentation bias.

### Sampling

#### Southern Subjects

The YRBS used a sampling and weighting procedure designed to obtain a representative sample of all public high school students in grades 9–12 in a southern state, with the exception of students in special education schools. The survey was previously determined to have adequate test-retest reliability [41] for six major areas of health risk behaviors: behaviors leading to intentional and unintentional injuries (e.g., violent, aggressive, or suicidal behaviors); use of tobacco, alcohol, and other drugs; sexual behaviors; dietary behaviors; and physical activity [42]. For the YRBS, the initial sampling frame consisted of 215 schools, stratified by enrollment size into three categories: small, medium, and large. Eighty-seven schools were randomly selected, and 63 agreed to participate (72% response rate).

Parent notification forms were distributed at least five days in advance of survey administration; parents who did not want their children to participate were required to return the form. Surveys were conducted by trained data collectors, who emphasized anonymity, privacy, and confidentiality. This research was approved by the referent university's review board for the rights of human subjects in research.

#### Midwestern Subjects

The 2003 national YRBS survey instrument was used to collect data on these adolescents, which was also determined to display adequate test-retest reliability recently [43]. Second period classes were randomly selected from each school until the total potential survey population reached approximately 10% of the total school student population. A total of 17 classes were selected to participate at two high schools (N = 244), of which 140 students participated (57% response rate).

Parent notification forms were distributed at least one week in advance of survey administration. However, unlike in the southern sample, parents who did want their children to participate were required to return to the form. The principal author of this paper along with trained data collectors who emphasized anonymity, privacy, and confidentiality collected all data. This research was approved by the referents university's review board for the rights of human subjects in research.

### Data analysis

With the southern sample, data analyses were conducted via SUDAAN [44], which accounts for the complex sampling design of the YRBS. Since the sampling design for the Midwestern sample was randomized, but not as complex as a statewide YRBS administration, all data analyses for the Midwestern sample were conducted with SAS (Cary, NC). Correlation analyses were performed to test the hypothesis that correlation coefficients for mental health (during the past 30 days) would correlate more strongly with self-rated health and QOL for both samples of adolescents when compared to physical health (during the past 30 days).

## Results

### Sample characteristics: Southern sample

There were a total of 5,220 observations in the 1997 YRBS. Respondents included 1,061 non-Hispanic white females (20.3%), 1,336 non-Hispanic black females (25.6%), 1,340 non-Hispanic white males (25.7%), 1,119 non-Hispanic black males (21.4%), 182 "other" females (3.5%) (Hispanic or Latino, Asian or Pacific Islander, American Indian or Alaskan Native), and 182 "other" males (3.5%). The "other" group was collapsed for reporting purposes.

As expected, the distribution of responses for each scale item was skewed toward the positive (Table 1). However, 785 (14.4%) adolescents still perceived their health as fair or poor, while 631 (11.4%) reported having 6 or more poor physical health days, and 1,291 (23.4%) reported having 6 or more poor mental health days.

**Table 1:** Responses to CDC scale items

	Southern Sample		Midwestern Sample	
**HRQOL item**	**Number**	**Percent**	**Number**	**Percent**

**Self-rated health**				
Excellent	1,088	19.96	22	15.7
Very good	1,652	30.30	39	27.9
Good	1,927	35.34	53	37.9
Fair	706	12.95	23	16.4
Poor	79	1.45	3	2.1
**Number of days physical health not good in past 30 days**				
0	2,229	40.41	48	34.3
1–2	1,739	31.53	51	36.5
3–5	917	16.62	24	17.2
6–9	339	6.15	9	6.4
10–19	160	2.90	3	2.1
20–29	41	0.74	2	1.4
30	91	1.65	3	2.1
**Number of days mental health not good in past 30 days**				
0	2,063	37.39	40	28.6
1–2	1,323	23.98	35	25.0
3–5	841	15.24	21	15.0
6–9	424	7.68	15	10.7
10–19	447	8.10	14	10.0
20–29	166	3.01	10	7.1
30	254	4.60	5	3.6

### Sample characteristics: Midwestern sample

There were a total of 140 observations in the Midwestern sample of adolescents. Respondents included 58 males (41.4%) and 82 females (58.6%). The majority of the sample described themselves as white (n = 127, 90.7%), while 13 students described themselves as non-white (9.3%).

As expected, the distribution of responses for each scale item was also skewed toward the positive (Table 1), and generally consistent with the southern sample. However, 26 (18.6%) adolescents still rated their health as fair or poor, while 17 (12.1%) reported having 6 or more poor physical health days, and 44 (31.4%) reported having 6 or more poor mental health days. Although the Midwestern sample reported poorer self-rated health and greater percentages of adolescents reporting poor physical and mental health days than the southern sample, similar trends are observed for each scale item between each sample, that is, both samples reported greater impairment in mental health days than physical health days, with the percentage of those who reported fair/poor self-rated health falling in between. Thus, these observations can be considered as validation of the general pattern of QOL reporting for this scale.

### Correlational analyses

#### Southern sample

**Figure 1:**
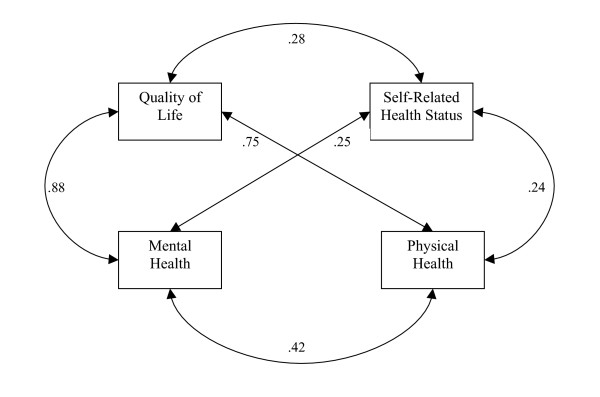
Relationship Between QOL and Self-Rated Health Status among Southern Adolescents.

Overall, correlation coefficients between self-rated health status and physical and mental health were modest, but significantly greater than zero at p < .0001 (Figure 1). The correlation coefficient between self-rated health status and the mean number of poor physical health days was slightly smaller (r = .24) than that between self-rated health status and the mean number of poor mental health days (r = .25). However, the correlation coefficient between the mean number of poor mental health days and the mean number of poor physical health days was larger (r = .42) than both coefficients with self-rated health status.

Since the correlation coefficients were similar between self-rated health status and physical and mental health, PROC GLM was employed to test whether physical or mental health was contributing in a greater degree to self-rated health status. It was hypothesized that if both variables were contributing equally to self-rated health status, then taking the difference between the two would not be significant. The Type III sums of squares (from PROC GLM) for the difference between physical and mental health (*F *= 4.408, P = .0295) suggest that although both physical and mental health contribute significantly to self-rated health status, significantly greater contributions are made from mental health for adolescents in this sample. However, the correlations between mean number of poor mental health days, mean number of poor physical health days, and self-rated health were still modest at best.

The next objective was to determine whether physical or mental health was contributing greater variance to QOL. Adolescent QOL ratings were more strongly correlated with mental health days(r = .88) than with physical health days (r = .75) (Figure 4). These results suggest that for this random sample of adolescents, while both physical and mental health are significant contributors to QOL, mental health (specifically mental health during the past 30 days) is a more significant correlate of QOL than physical health (during the past 30 days).

#### Midwestern sample

**Figure 2:**
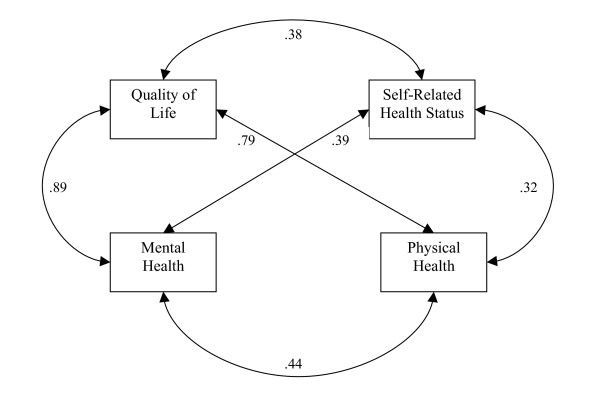
Relationship Between QOL and Self-Rated Health Status among Midwestern Adolescents.

All correlation coefficients were significant in the Midwestern sample (p < .0001), suggesting that sample size was not a significant contributing factor to the significant observed correlations between all scale items in the southern sample. Correlation coefficients between self-rated health status and the mean number of poor physical and mental health days were still moderate, but significantly greater than zero at p < .0001, and larger than those observed among the southern sample (Figure 2). In addition, the correlation patterns between self-rated health status and physical health (r = .32), and self-rated health status and mental health (r = .39), was consistent with the southern sample. However, the greater contributions of mental health to self-rated health, as opposed to physical health are more clearly defined in the Midwestern sample. Also consistent with the southern sample, the correlation coefficient between the mean number of poor mental health days and the mean number of poor physical health days was larger (r = .44) than both coefficients with self-rated health status.

Results obtained from the southern sample regarding the strength of the correlation coefficients between mean number of poor physical health days and the mean number of poor mental health days and QOL were also duplicated in the Midwestern sample. In this sample, adolescent QOL ratings were more strongly correlated with mental health days (r = .89) than with physical health days (r = .79) (Figure 2). These results suggest that for this random sample of adolescents, while both physical and mental health are significant contributors to QOL, mental health (specifically mental health during the past 30 days) is a more significant predictor of QOL than physical health (during the past 30 days). Furthermore, although the strength of correlations differ slightly in strength between the southern and Midwestern samples, both models are consistent in their findings that mental health appears to be a greater contributor to self-rated health and QOL among adolescents.

## Discussion

The correlation coefficients between the mean number of poor mental health days and the mean number of poor physical health days was larger in both the southern (r = .42) and Midwestern (r = .44) samples than both coefficients with self-rated health status in these analyses, which is consistent with Smith's et al. [12] findings. In the adult QOL literature utilizing this same scale, the mean number of poor mental health days was more highly correlated with the mean number of poor physical health days (r = 0.66) [45], suggesting that adolescents view physical and mental health with a greater degree of separation than adults. In addition, when rating QOL, although both physical and mental health are important evaluations for adolescents, mental health appears to be a greater contributor, which is consistent with what has been observed among adults [12].

It is important to understand whether mental or physical health evaluations contribute more heavily to adolescent QOL. In this study, the contributions of mental health days to QOL exceeded the contributions of physical health days (r = .88 and r = .75, respectively in the southern sample; r = .89 and r = .79, respectively in the Midwestern sample) for adolescents, which can better assist health practitioners, counselors, medical personnel, researchers, and other human service personnel working with adolescents and in program resource allocation. However, contrary to adults with chronic disease [12], adolescent self-rated health status in these samples is based more strongly on mental health and to a lesser extent on physical health. These findings suggest mental health issues are more salient among adolescents than among adults, whether rating their QOL or health status, and adolescent QOL is significantly less likely to be improved if only self-rated health status indicators are utilized in research designs. Therefore, if *Healthy People 2010 *objectives are going to be attained among adolescents, efforts to improve mental health need more emphasis on health promoting efforts and in clinical applications.

Secondary findings generated by this study are the correlation strengths among the variables analyzed. Although similar results were obtained between QOL and mental and physical health as in other investigations [12], the modest to moderate correlation coefficients between self-rated health and QOL (r = .28 in the southern sample; r = .38 in the Midwestern sample) and correspondingly strong coefficients between QOL and both physical health days and mental health days, this study provides additional evidence that adolescents may be evaluating two different constructs, as was concluded by Smith et al. [12] and recently by Zullig et al [32]. These results suggest that although self-rated health appears to be a component of adolescent QOL assessment, adolescents may be accounting for other factors other than mental or physical health days when self-rating their health. However, what these other factors are when adolescents are determining their self-rated health remains largely unexplained by these results. Evidence garnered from this preliminary study suggests that adolescents are clearly giving only modest attention to physical and mental health status when self-rating their health, but not when determining their QOL.

These findings carry important implications for selecting instruments for QOL research among adolescents. First, caution needs to be exercised when choosing adolescent QOL instruments. Our results suggest that self-rated health status measures should not be construed exclusively as QOL assessments. As an example, Huang et al [23] used the standard ordinal-scaled self-rated health measure as their outcome measure of quality of life in their sample of adolescents with diabetes. These results suggest that Huang and colleagues may have misrepresented their QOL study findings substantially because better self-rated health does not necessarily equate to increased quality of life. Thus, favorable intervention effects on self-rated health status may be significantly less effective for QOL among adolescents. Based on these results, QOL, in the context of public high school adolescents, is the subjective appraisal of one's current life based largely upon mental health and to a lesser extent on physical health.

## Limitations

One limitation to this study is not having a concrete measure of social functioning for these samples of adolescents. Although one can conjecture the importance of including a measure of social functioning, without a measure(s) of social functioning, it is difficult to ascertain what other possible domains adolescents are rating when determining their self-rated health and the potential contributions of social functioning to mental health. Second, although these items from the CDC scale have performed preliminarily well in validity analyses with adolescents [32], scale reliability has not been tested. Third, a response rate of only 57% was obtained for Midwestern sample, which may have biased the results. Active consent procedures utilized in the Midwestern sample likely decreased this sample size. However, owing to a clear pattern of findings across both samples, it is unlikely any significant sampling bias occurred.

This study also has several study strengths. First, this study utilized two very different samples based on racial composition, sample timing (1997 & 2003), and geography (the south vs. midwest). Second, the Midwestern sample (N = 140) was much smaller than the southern sample (N = 5,220), yet the correlation patterns are both significant and similar in each sample. The fact that the Midwestern sample retained the significance levels observed among the much larger southern sample alleviates any concern that sample size is driving significance.

## Conclusion

The general correlational patterns observed among these two geographically different and racially diverse study populations warrants attention. First, similar to adults, adolescent QOL determinants appear to be primarily related to mental and physical health. Second, although mental and physical health both contribute significantly to adolescent self-rated health, mental health appears to make greater contributions, which is opposite what has been observed with adults. However, adolescents may also be considering other health-related constructs in addition to their physical and mental functioning when self-rating their health. Coping styles, social support, social bonding, and personality characteristics are only a few possible mediators that may be considered. In this regard, further research is needed with a more comprehensive approach to self-rated health for teenagers. Finally, the separation of QOL and self-rated health measures appears to be justified by these analyses, as has been posited in adult QOL research.

## Authors' contributions

KJZ conceived and designed the study, collected and analyzed the Midwestern state data, and coordinated all aspects of the manuscript. RFV helped draft the manuscript and was Principal Investigator from the southern state YRBS. JWD formatted and performed statistical analyses on the southern state data. All authors read and approved the final manuscript.
